# To each his own: no evidence of gyrodactylid parasite host switches from invasive poeciliid fishes to *Goodea atripinnis* Jordan (Cyprinodontiformes: Goodeidae), the most dominant endemic freshwater goodeid fish in the Mexican Highlands

**DOI:** 10.1186/s13071-016-1861-2

**Published:** 2016-11-25

**Authors:** Miguel Rubio-Godoy, Ulises Razo-Mendivil, Adriana García-Vásquez, Mark A. Freeman, Andrew P. Shinn, Giuseppe Paladini

**Affiliations:** 1 Instituto de Ecología, A.C., Red de Biología Evolutiva, Km 2.5 Ant. Carretera a Coatepec, Xalapa, Veracruz 91070 Mexico; 2 Ross University School of Veterinary Medicine, PO Box 334, Basseterre, St. Kitts And Nevis; 3 Institute of Aquaculture, Faculty of Natural Sciences, University of Stirling, FK9 4LA Stirling, UK; 4 Fish Vet Group Asia Limited, 99/386, Chaengwattana Building, Moo 2, Chaengwattana Rd., Kwaeng Toongsonghong, Khet Laksi, Bangkok, 10210 Thailand

**Keywords:** Monogenea, *Gyrodactylus tomahuac*, *Gyrodactylus lamothei*, ITS, Invasive species, Enemy release hypothesis, Mexico

## Abstract

**Background:**

Goodeid topminnows are live-bearing fishes endemic to the Mexican Highlands (Mesa Central, MC). Unfortunately, in the MC, environmental degradation and introduced species have pushed several goodeid species to the brink of extinction. Invasive fishes can introduce exotic parasites, and the most abundant goodeid, blackfin goodea *Goodea atripinnis* Jordan, is parasitised by six exotic helminths. Poeciliids are widely dispersed invasive fishes, which exert negative ecological effects on goodeids. Poeciliids host several species of the monogenean genus *Gyrodactylus* von Nordmann, 1832, including pathogenic, invasive parasites. Here, we looked for evidence of *Gyrodactylus* species switching hosts from poeciliids to goodeids.

**Methods:**

Fish were collected in rivers draining the MC into both sides of the continental divide. Hosts were screened for gyrodactylid parasites in localities where *G. atripinnis* and poeciliids occurred sympatrically. *Gyrodactylus* specimens were characterised morphologically (attachment apparatus) and molecularly (internal transcribed spacer region, ITS). A Bayesian phylogenetic tree using ITS sequences established relationships between gyrodactylids collected from goodeid fishes and those from parasites infecting poeciliids.

**Results:**

Gyrodactylids were collected from *G. atripinnis* in six localities on both sides of the watershed where exotic poeciliids occurred sympatrically. Morphological and molecular analyses indicated the presence of four undescribed species of *Gyrodactylus* infecting this goodeid host. *Gyrodactylus tomahuac* n. sp., the most abundant and geographically widespread species, is described here. The other three *Gyrodactylus* spp. are not described, but their ITS sequences are used as molecular data presented here, are the only available for gyrodactylids infecting goodeid fishes. Morphological and molecular data suggest that two distinct groups of gyrodactylids infect goodeids, one of which shares a common ancestor with gyrodactylids parasitizing poeciliids.

**Conclusions:**

No evidence was found of gyrodactylids switching hosts from invasive poeciliids to endemic goodeids, nor vice versa. Moreover, considering that *G. atripinnis* is known to host both *Gyrodactylus lamothei* Mendoza-Palmero, Sereno-Uribe & Salgado-Maldonado, 2009 and *Gyrodactylus mexicanus* Mendoza-Palmero, Sereno-Uribe & Salgado-Maldonado, 2009, with the addition of *G. tomahuac* n. sp. and the three undescribed *Gyrodactylus* spp. reported, at least six gyrodactylids may infect this host. This would make monogeneans the second most abundant parasite group infecting *G. atripinnis*, which to date is known to harbour 22 helminth species: nine digeneans, five nematodes, four cestodes, three monogeneans and one acanthocephalan.

## Background

Human-mediated introduction of non-native (also referred to as alien or exotic) fishes has resulted in the establishment of breeding populations of exotic fishes in freshwater water bodies worldwide [1–4]. Introduced fishes may compete directly with native fishes, and several invasive fish species have been demonstrated to exert negative ecological effects following invasion [1–3, 5]. Alien fishes can additionally act as carriers of non-indigenous parasites, which can negatively affect native hosts, as has been shown for several flatworm parasites of the class Monogenea Carus, 1863, whose short, one-host direct life-cycle facilitates establishment and invasion. Two compelling examples involve monogeneans from the genus *Gyrodactylus* von Nordmann, 1832 introduced along with their commercially-important fish hosts: *Gyrodactylus salaris* Malmberg, 1957, introduced in the mid-1970’s from the Baltic Sea to Norway, which has caused a highly pathogenic epizootic among wild and farmed salmonid fishes [6]; and *Gyrodactylus cichlidarum* Paperna, 1968, a parasite which has achieved almost global distribution through the translocation of its “tilapia” (*Oreochromis* spp.) hosts and has caused mass mortalities of cultured fish stocks [7].

The ornamental fish trade is an important component of international commerce, with over 1.5 billion fish belonging to more than 4000 freshwater and 1400 marine species traded annually and generating revenue in excess of US $ 6 billion [8]. As is the case for species translocated for aquacultural purposes, anthropogenic introduction of exotic ornamental fishes has resulted in alien monogenean parasites causing epizootic outbreaks in confined fish populations, for instance in display aquaria [9]. Furthermore, monogeneans co-introduced to continental freshwater bodies with their exotic fish hosts can contribute to the loss of native fish biodiversity, following parasite host switches (HS) from the introduced, alien fish to indigenous fish hosts (reviewed in [10]). One group of ornamental fishes exemplifies the invasiveness of feral aquarium fishes and their role as vectors to monogenean parasites: the small, live-bearing freshwater fishes belonging to the cyprinodontiform family Poeciliidae, which were originally distributed in the Americas, Africa and Madagascar [11]. Poeciliids have been translocated worldwide with the aquarium trade (e.g. “guppies” and “mollies” from the genus *Poecilia* Bloch & Schneider, “swordtails” and “platys” from the genus *Xiphophorus* Heckel) and mosquito control programmes (“mosquito fish” from the genera *Gambusia* Poey and *Poecilia*), and now introduced, feral populations of exotic poeciliids are established in all continents except Antarctica [3]. Arguably, poeciliids are the most successful exotic fish family in Australia [12], a country with strictly enforced quarantine rules governing the import of live animals, with five ornamental species having established breeding populations in the continent: Eastern mosquito fish, *Gambusia holbrooki* Girard, green swordtail, *Xiphophorus hellerii* Heckel, Southern platyfish, *Xiphophorus maculatus* (Günther), guppy, *Poecilia reticulata* Peters, and dusky millions fish, *Phalloceros caudimaculatus* (Hensel) [13]. The same five poeciliid species plus mosquito fish, *Gambusia affinis* (Baird & Girard), have established reproductively in Indian continental waters [10]. In both cases, exotic poeciliid fishes have co-introduced their gyrodactylid parasites [10, 13]; nonetheless, no evidence was found in Australia of gyrodactylids switching hosts from exotic poeciliid to native fishes. Trade of ornamental poeciliids has also contributed to the wide dissemination of their monogenean parasites: several gyrodactylid species have been recorded to infect aquarium fishes in Canada, UK, Czech Republic, Singapore, Korea, India, Australia, etc. [10, 12, 14]. In fact, five of the 19 species of *Gyrodactylus* known to infect poeciliid fishes were originally obtained from exotic fishes reared in aquaria (reviewed in [14]). Although their potential pathogenic effects on native fish populations have not been fully demonstrated, some gyrodactylids known to infect poeciliid fishes are remarkable for the extent of their geographical distribution following anthropogenic translocation, and for the range of host species they infect. For instance, *Gyrodactylus bullatarudis* Turnbull, 1956 has been recorded from six poeciliid fish species from the genera *Gambusia*, *Poecilia*, *Pseudoxiphophorus* Bleeker (syn. *Heterandria*), and *Xiphophorus*, infecting wild, captive and feral populations in North America, Europe and Australia (reviewed in [14]), and has also been shown to infect the non-poeciliid, cyprinodontiform killifish, *Anablepsoides hartii* (Boulenger) (syn. *Rivulus hartii*) [15].

Poeciliid fishes are native to the southern, Neotropical regions of Mexico, but have been extensively introduced to the central and northern, Nearctic highlands (Mesa Central, MC) of the country [4, 5, 16], where endemic goodeid fishes occur. Thus, mosquito fish, *G. affinis*, originally from north-eastern Mexico and the USA, has been introduced to the MC; as well as several poeciliid species originally restricted to the Gulf of Mexico watershed, such as shortfin molly *Poecilia mexicana* Steindachner, porthole livebearer *Poeciliopsis gracilis* (Heckel), two-spot livebearer *Pseudoxiphophorus bimaculatus* Heckel (syn. *Heterandria bimaculata*), green swordtail *X. hellerii*, and variable platyfish *Xiphophorus variatus* (Meek) [4, 17], and invasive Trinidadian guppies *Poecilia reticulata* Peters [2, 4, 18].

Goodeid topminnows or splitfins are a family of live-bearing cyprinodontoid fishes that evolved in the MC, and occur in Central Mexico and North America [17]; the subfamilies Goodeinae and Empetrichthyinae are principally distributed across these two geographic regions, respectively [19]. About 45 species from the MC are classified within the sub-family Goodeinae [20]. Goodeines were originally distributed in drainages along both the Pacific Ocean and Gulf of Mexico slopes; unfortunately, the MC is one of the most densely populated regions in Mexico and habitat destruction, water pollution, and introduction of exotic species have pushed several endemic fishes to the brink of extinction, with some goodeid species effectively extirpated from their natural habitats [21]. Only two goodeid species distributed in Central Mexico can be considered to be in no conservation risk category: the blackfin goodea *Goodea atripinnis* Jordan, and the dark-edged splitfin *Girardinichthys multiradiatus* (Meek) [22]. *Goodea atripinnis* remains common in many areas and is probably still the most abundant goodeid species overall, but its distribution and abundance have steadily declined over the last 30 years [20, 21, 23].

*Goodea atripinnis* has been recorded to be infected by 22 helminth parasite species [24], including 16 native and six alien parasite taxa: nine digeneans, five nematodes, four cestodes, three monogeneans and one acanthocephalan. Of these, the following are invasive, exotic parasites [18, 24]: the monogeneans *Cichlidogyrus sclerosus* Paperna & Thurston, 1969 and *Dactylogyrus extensus* Mueller & Van Cleave, 1932, the digenean *Centrocestus formosanus* Nishigori, 1924, the cestodes *Schyzocotyle* (syn. *Bothriocephalus*) *acheilognathi* (Yamaguti, 1934) Brabec, Waeschenbach, Scholz, Littlewood & Kuchta, 2015 and *Proteocephalus ambloplitis* (Leidy, 1887), and the nematode *Pseudocapillaria tomentosa* (Dujardin, 1843). Only three species of most likely indigenous monogeneans have been formally described from Mexican goodeids, all of which are known to infect *G. atripinnis*: the dactylogyrid *Salsuginus angularis* (Mueller, 1934) Beverley-Burton, 1984, and the gyrodactylids *Gyrodactylus lamothei* Mendoza-Palmero, Sereno-Uribe & Salgado-Maldonado, 2009 and *Gyrodactylus mexicanus* Mendoza-Palmero, Sereno-Uribe & Salgado-Maldonado, 2009 (Table 1), plus several records of undescribed species of *Gyrodactylus* from 11 goodeid fish species (Table 2). To date, 22 valid *Gyrodactylus* species are known to infect native and introduced fishes in Mexico (Table 1), including 11 species infecting poeciliid fishes [14, 25]. Some of the species of *Gyrodactylus* recorded in the country are capable of infecting several different genera of fish hosts (Table 1). For instance, both *G. lamothei* and *G. mexicanus* have been recorded on goodeids from the genera *Allotoca*, *Girardinichthys* and *Goodea* [24]; and *Gyrodactylus pseudobullatarudis* García-Vásquez, Razo-Mendivil & Rubio-Godoy, 2015 and *Gyrodactylus xtachuna* García-Vásquez, Razo-Mendivil & Rubio-Godoy, 2015 are able to infect poeciliids from the genera *Poecilia*, *Poeciliopsis*, *Pseudoxiphophorus* (syn. *Heterandria*) and *Xiphophorus* [14], which overlaps with the known host range of *G. bullatarudis* (see above)*.*

**Table 1:** Species of *Gyrodactylus* von Nordmann, 1832 recorded from Mexican freshwater fishes

*Gyrodactylus* spp.	Host (Family)	Locality	Reference
*G. actzu* García-Vásquez, Razo-Mendivil & Rubio-Godoy, 2015	*Poecilia mexicana* Steindachner (Poeciliidae)	Ver	[14]
*G. apazapanensis* García-Vásquez, Razo-Mendivil & Rubio-Godoy, 2015	*P. mexicana* (Poeciliidae); *Xiphophorus hellerii* Heckel (Poeciliidae)	Ver	[14]
*G. bullatarudis* Turnbull, 1956	*Pseudoxiphophorus bimaculatus* (syn*. Heterandria bimaculata*) Heckel (Poeciliidae)	Ver	[46]
*G. cichlidarum* Paperna,1968	*Oreochromis mossambicus* (Peters) (Cichlidae)	Sin, Tab, Ver, Yuc	[7, 47]
	*Oreochromis niloticus* (L.) (Cichlidae)	Sin, Tab, Ver, Yuc	[7, 47–49]
	Rocky Mountain strain^a^	Ver	
	Pargo UNAM strain^b^	Ver	
	Florida red strain^c^	Ver	
*G. elegans* von Nordmann, 1832^d^	*Girardinichthys multiradiatus* (Meek) (Goodeidae)	Mex	[50, 51]
*G. jarocho* Rubio-Godoy, Paladini, García-Vásquez & Shinn, 2010	*X. hellerii* (Poeciliidae)	Ver	[25]
*G. lamothei* Mendoza-Palmero, Sereno-Uribe & Salgado-Maldonado, 2009	*Allotoca diazi* (Meek) (Goodeidae) *Allotoca dugesii* (Bean) (Goodeidae) *G. multiradiatus* (Goodeidae) *Goodea atripinnis* Jordan (Goodeidae)	Mich Mich DF, Mex Qro	[24, 26]
*G. lhkahuili* García-Vásquez, Razo-Mendivil & Rubio-Godoy, 2015	*P. mexicana* (Poeciliidae)	Ver	[14]
*G. mexicanus* Mendoza-Palmero, Sereno-Uribe & Salgado-Maldonado, 2009	*A. dugesii* (Goodeidae) *G. multiradiatus* (Meek) (Goodeidae) *G. atripinnis* (Goodeidae) *Skiffia lermae* Meek (Goodeidae) *Xenotoca variata* (Bean) (Goodeidae)	Mich Mex DF, Gto DF Gto	[24, 26, 52]
*G. microdactylus* García-Vásquez, Razo-Mendivil & Rubio-Godoy, 2015	*P. mexicana* (Poeciliidae)	Ver	[14]
*G. neotropicalis* Kritsky & Fritts, 1970	*Astyanax fasciatus* (Cuvier) (Characidae)	Yuc	[53]
*G. niloticus* Cone, Arthur & Bondad-Reantaso, 1995 (junior synonym of *G. cichlidarum*, see García-Vásquez et al. 2007)	*Oreochromis aureus* (Steindachner) (Cichlidae) *O. mossambicus* (Cichlidae) *O. niloticus* (Cichlidae)	Tab Tab Tab	[50, 54, 55]
*G. pakan* Razo-Mendivil, García-Vásquez & Rubio-Godoy, 2016	*Astyanax aeneus* (Günther) (Characidae)	Ver	[56]
*G. pseudobullatarudis* García-Vásquez, Razo-Mendivil & Rubio-Godoy, 2015	*P. mexicana* (Poeciliidae) *Poeciliopsis gracilis* (Heckel) (Poeciliidae) *X. hellerii* (Poeciliidae)	Hgo, Ver Pue Ver	[14, 25]
*G. salmonis* (Yin & Sproston, 1948)	*Oncorhynchus mykiss* (Walbaum) (Salmonidae)	Ver	[33]
*G. spathulatus* Mueller, 1936	*Catostomus nebuliferus* Garman (Catostomidae)	Dgo	[57]
	*Gila conspersa* Garman (Cyprinidae)	Dgo	
	*Ictalurus* cf. *pricei* (Rutter) (Ictaluridae)	Dgo	
*G. takoke* García-Vásquez, Razo-Mendivil & Rubio-Godoy, 2015	*P. bimaculatus* (*=H. bimaculata*) (Poeciliidae) *P. gracilis* (Poeciliidae)	Ver Pue, Ver	[14]
*G. teken* Razo-Mendivil, García-Vásquez & Rubio-Godoy, 2016	*Astyanax aeneus* (Günther) (Characidae)	Ver	[56]
*G. unami* García-Vásquez, Razo-Mendivil & Rubio-Godoy, 2015	*P. gracilis* (Poeciliidae)	Ver	[14]
*G. xalapensis* Rubio-Godoy, Paladini, García-Vásquez & Shinn, 2010	*P. bimaculatus* (*=H. bimaculata*) (Poeciliidae)	Ver	[25]
*G. xtachuna* García-Vásquez, Razo-Mendivil & Rubio-Godoy, 2015	*P. gracilis* (Poeciliidae) *P. mexicana* (Poeciliidae) *P. bimaculatus* (*=H. bimaculata*) (Poeciliidae) *X. hellerii* (Poeciliidae)	Pue, Ver Ver Ver Ver	[14, 25]
*G. yacatli* García-Vásquez, Hansen, Christison, Bron & Shinn, 2011	*O. niloticus* (Cichlidae)	Sin, Tab	[58]

*Abbreviations*: *DF* Mexico City, *Dgo* Durango, *Gto* Guanajuato, *Hgo* Hidalgo, *Mex* Estado de México, *Mich* Michoacán, *Pue* Puebla, *Qro* Querétaro, *Sin* Sinaloa, *Tab* Tabasco, *Ver* Veracruz, *Yuc* Yucatán

^a^Pargo-UNAM is an *Oreochromis* hybrid, whose genetic composition is: 50% Florida red tilapia, 25% Rocky Mountain tilapia and 25% red *O. niloticus*

^b^Rocky mountain is an *Oreochromis* hybrid, whose genetic composition is: 50% *O. niloticus* and 50% *O. aureus*

^c^Florida red tilapia is an *Oreochromis* hybrid, whose genetic composition is: 50% *O. mossambicus* and 50% *O. aureus*

^d^This species was identified by Salgado-Maldonado et al. [50] and Sánchez-Nava et al. [51] as *Gyrodactylus* cf. *elegans* von Nordmann, 1832; however, re-examination of the samples suggests that this material represents a further two undescribed species (data not shown, pers. obs.)

**Table 2:** Undescribed *Gyrodactylus* spp. recorded from different families of freshwater fishes in Mexico

Host^a^	Locality	Reference
Catostomidae		
*Catostomus nebuliferus* Garman	Dgo	[57]
Centrarchidae		
*Lepomis macrochirus* Rafinesque	Dgo	[57]
Characidae		
*Astaynax aeneus* (Günther)	Oax	[54]
*Astyanax mexicanus* (De Filippi)	Dgo, Qro	[57, 59]
Cichlidae		
*Cichlasoma geddesi* (Regan)	Yuc	[60]
*Herichthys cyanoguttatus* Baird & Girard	Hgo	[25]
*Herichthys deppii* (Heckel)	Ver	[25]
*Herichthys pantostictus* (Taylor & Miller)	Hgo	[25]
*Oreochromis aureus* (Steindachner)	Qro	[61]
*Oreochromis mossambicus* (Peters)	Son	[62]
*Oreochromis* sp*.*	Mor	[63]
*Parachromis managuensis* (Günther)	Camp	[60]
*Paraneetroplus fenestratus* (Günther)	Ver	[54]
*Thorichthys aureus* (Günther)	Yuc	[60]
*Thorichthys helleri* (Steindachner)	Tab	[60]
*Thorichthys meeki* Brind	Camp	[60]
Cyprinidae		
*Aztecula sallaei* (Günther)	Qro	[61]
*Campostoma ornatum* Girard	Dgo	[57, 59, 64]
*Carassius auratus* (L.)	DF, Mex, Mor	[65, 66]
*Cyprinodon atrorus* Miller	Coah	[67]
*Cyprinus carpio* (L.)	Hgo, Mor, Qro, Ver	[61, 65, 66, 68]
*Gila conspersa* Garman	Dgo	[57]
*Notropis boucardi* (Günther)	Mor	[69]
*Notropis nazas* Meek	Dgo	[57]
*Notropis* sp*.*	Mexico - no precise location given	[70]
*Pimephales promelas* Rafinesque	Dgo	[57]
Eleotridae		
*Dormitator maculatus* (Bloch)	Ver	[54]
*Gobiomorus dormitor* Lacépède	Ver	[54]
Goodeidae		
*Allotoca diazi* (Meek)	Mich	[24]
*Allotoca dugesii* (Bean)	Mich	[24]
*Characodon audax* Smith & Miller	Dgo	[24]
*Characodon lateralis* Günther	Dgo	[24]
*Girardinichthys multiradiatus* (Meek)	Mex, Mor, Oax	[24, 50, 51, 54, 66]
*Girardinichthys viviparus (*Bustamante)	Hgo	[71]
*Goodea atripinnis* Jordan	Gto, Jal, Mex, Mich, Qro	[24, 52, 61] Present study
*Skiffia lermae* Meek	Mich	[24]
*Xenotoca melanosoma* Fitzsimons	Jal	[24]
*Xenotoca variata* (Bean)	Qro	[61]
*Zoogoneticus quitzeoensis* (Bean)	Mich	[24]
Heptateridae		
*Rhamdia quelen* (Quoy & Gaimard)	Ver	[54]
Ictaluridae		
*Ictalurus punctatus* (Rafinesque)	Tamps	[65, 68]
Poeciliidae		
*Gambusia yucatana* Regan	Yuc	[53, 72]
*Poecilia mexicana* Steindachner	Mex, Oax, Qro, Ver	[54, 73] Present study
*Poecilia reticulata* Peters	Qro	Present study
*Poecilia sphenops* Valenciennes	Nay	[50]
*Poeciliopsis gracilis* (Heckel)	Mor, Oax, Ver	[52, 54, 73] Present study
*Poeciliopsis infans* (Woolman)	Gto, Jal, Mich	[50, 52] Present study
*Pseudoxiphophorus jonesii* (Günther)	Qro	Present study
*Pseudoxiphophorus bimaculatus* Heckel	Ver	[52]
*Xiphophorus hellerii* Heckel	Ver	Present study
Profundulidae		
*Profundulus balsanus* Ahl	Oax	[74]
*Profundulus punctatus* (Günther)	Oax	[75]
Salmonidae		
*Oncorhynchus mykiss* (Walbaum)	DF, Mex	[65, 68]

*Abbreviations*: *Camp* Campeche, *Coah* Coahuila, *DF* Mexico City, *Dgo* Durango, *Hgo* Hidalgo, *Jal* Jalisco, *Mex* Estado de México, *Mich* Michoacán, *Mor* Morelos, *Nay* Nayarit, *Oax* Oaxaca, *Pue* Puebla, *Qro* Querétaro, *Sin* Sinaloa, *Son* Sonora, *Tab* Tabasco, *Tamps* Tamaulipas, *Ver* Veracruz, *Yuc* Yucatán

^a^Valid fish names checked in FishBase, August 2016 [11]; except genus *Pseudoxiphophorus* (syn. *Heterandria*), which has not been updated

Poeciliids and goodeids are morphologically similar viviparous, cyprinodontiform fishes that exhibit comparable habitat use [17], and are now sympatric following the human-mediated translocation of poeciliids to the MC. Exotic poeciliids have been shown to modify the ecological structure, function and native species abundance of water bodies following invasion [1], which is usually attributed to competition for habitat and/or food [3]. Additionally, invasive fishes may prey on native species, as shown for *Xiphophorus* spp., which feed on eggs and juveniles and have been implicated in the extinction of the goodeids golden skiffia, *Skiffia francesae* Kingston, and banded allotoca, *Allotoca goslinei* Smith & Miller [5]. A further negative impact of invasive poeciliids that has been recorded in the MC is the heterospecific harassment of native goodeids; in particular, male guppies (*P. reticulata*) have been recorded to attempt forced copulations of female twoline skiffia *Skiffia bilineata* (Bean) [2].

Given that the parasite fauna of the relatively abundant and widely distributed blackfin goodea, *G. atripinnis* is well known [24], with the exception of the Monogenea, and considering that *Gyrodactylus* sp. has been recorded to infect this goodeid in several states located in central Mexico (Table 2), a first objective of this study was to characterise gyrodactylids infecting this host in the MC. This included trying to collect specimens of *G. lamothei* and *G. mexicanus*, to characterise them molecularly, as their description contained morphometric data only [26]. A second objective was to ascertain whether any species of *Gyrodactylus* had switched host from introduced, alien poeciliids to endemic *G. atripinnis*. This was considered given that, as mentioned above, some species of *Gyrodactylus* have been shown to be extremely invasive following co-introduction with their fish hosts; and that poeciliids and goodeids are now sympatric in much of the MC and are likely to interact as they are morphologically and ecologically similar. We hypothesised HS were more likely from poeciliid to goodeid fishes than vice versa, because the first are very successful invaders and generally outnumber the endemic, endangered goodeids [4, 16], although evidently HS could occur in both directions. Potential HS from poeciliids to goodeids could in principle be recognised, considering that all 19 gyrodactylid species known to infect poeciliid fishes have been characterised morphologically [25], and parts of the genome have been sequenced for others [14]. To test the HS hypothesis, several localities on rivers draining the MC into both the Gulf of Mexico and the Pacific Ocean were sampled, and gyrodactylid parasites recovered from both *G. atripinnis* and poeciliids were analysed by morphological and molecular-based approaches from those localities where this goodeid fish and poeciliid fishes were found in sympatry. Finally, analysing the gyrodactylid fauna of non-native poeciliid fishes in the MC enabled a preliminary assessment of the enemy release hypothesis, which posits that upon translocation, introduced species lose some of their natural enemies such as parasites, thereby gaining a fitness advantage - a topic which was recently addressed studying the *Gyrodactylus* fauna of native and introduced minnow populations in Norway [27].

## Methods

### Specimen collection

Fishes were collected by electrofishing at different localities in the Río Pánuco and the Río Lerma basins, which flow from the MC into the Gulf of Mexico and the Pacific Ocean, respectively. Different species of goodeid [*G. atripinnis*, *G. multiradiatus*, *S. bilineata* and *Xenotoca variata* (Bean)] and poeciliid [*P. bimaculatus*, *P. gracilis*, *P. mexicana*, *P. reticulata*, *Poeciliopsis infans* (Woolman) and *Pseudoxiphophorus jonesii* (Günther)] fishes were collected. In the present study, only precise information is given for localities where blackfin goodea, *G. atripinnis* and poeciliid fish species were collected simultaneously (Table 3). Specimens were collected in April 2008 in the Río Moctezuma, Vega de Ramírez, Querétaro (21°03′31.01″N, 99°28′03.68″W); and in May 2014, in streams in Araro, Michoacán (19°54′27.52″N, 100°50′23.36″W); El Fresno, Guanajuato (20°16′39.07″N, 100°29′09.69″W); San Miguel Tlaxcaltepec (20°06′23.34″N, 100°07′36.74″W) and Santiago Mezquititlán (20°04′37.01″N, 100°04′29.38″W), both in Querétaro; in a reservoir at San Nicolás Peralta, Estado de México (19°21′26.75″N, 99°29′38.66″W); and along the northern shore of Lago de Chapala, Chapala, Jalisco (20°17′18.4″N, 103°11′35.9″W). Live fish were kept in buckets fitted with battery-operated aerators whilst electrofishing; an effort was made to keep poeciliid and goodeid species in separate buckets, to reduce the possibility of gyrodactylid parasite HS between non-related, cohabiting fishes [28]. After euthanasia, fish were preserved in groups separated by species and locality, and placed in labelled plastic bottles containing 96% ethanol until they were screened microscopically.

**Table 3:** *Gyrodactylus* spp. infection data for localities where *Goodea atripinnis* occurs in sympatry with poeciliid fishes

Locality	Fishes collected		*Gyrodactylus* infection on *G. atripinnis* (prevalence; mean abundance)	*Gyrodactylus* spp. recorded on *G. atripinnis*	Analytical method (specimens analysed)		*Gyrodactylus* spp. recorded on poeciliid species
	Goodeid species	Poeciliid species			Morphology	Sequencing	
Río Pánuco basin							
Vega de Ramírez, Qro.	*Goodea atripinnis* (*n* = 10)	*Poecilia mexicana* (*n* = 87)	100%; 4.1 worms/host	*G. tomahuac* n. sp.	9	4	*P. mexicana*: *G. pseudobullatarudis*, *G. xtachuna*;
		*Poecilia reticulata* (*n* = 17)					*P. reticulata*: *Gyrodactylus* sp.
Río Lerma basin							
Araro, Mich.	*Goodea atripinnis* (*n* = 7)	*Poecilia mexicana* (*n* = 9)	14%; 0.4 worms/host	*G. tomahuac* n. sp.	1	1	*P. mexicana*: *G. cichlidarum*;
	*Skiffia bilineata* (*n* = 2)	*Poeciliopsis infans* (*n* = 500+)					*P. bimaculatus*: *G. cichlidarum*;
	*Xenotoca variata* (*n* = 1)	*Pseudoxiphophorus bimaculatus* (*n* = 7)					*P. infans*: *Gyrodactylus* sp.
El Fresno, Gto.	*Goodea atripinnis* (*n* = 1)	*Pseudoxiphophorus bimaculatus* (*n* = 6)	100%; 7 worms/host	*G. tomahuac* n. sp.	4	4	
				*Gyrodactylus* sp. 3	1^a^	1	
San Miguel Tlaxcaltepec, Qro.	*Goodea atripinnis* (*n* = 23)	*Pseudoxiphophorus jonesii* (*n* = 49)	26%; 2.7 worms/host	*G. tomahuac* n. sp.	1	1	*P. jonesii*: *Gyrodactylus* sp.
				*Gyrodactylus* sp. 3	3^a^	3	
Lago de Chapala, Jal.	*Goodea atripinnis* (*n* = 4)	*Poeciliopsis infans* (*n* = 20)	25%; 0.3 worms/host	*Gyrodactylus* sp. 1	1^a^	1	
Santiago Mezquititlán, Qro.	*Goodea atripinnis* (*n* = 1)	*Poecilia mexicana* (*n* = 5)	100%; 5 worms/host	*Gyrodactylus* sp. 2	2^a^	2	

*Abbreviations*: *Qro* Querétaro, *Mich* Michoacán, *Gto* Guanajuato, *Jal* Jalisco

^a^Illustrative drawings are provided but complete descriptions will be dealt with elsewhere following analysis of further specimens

### Specimen preparation

Fish fins, body and gills were inspected under a dissection microscope, and parasites found were dislodged gently using acupuncture needles. Representative gyrodactylid specimens were prepared as whole mounts in ammonium picrate glycerine following the procedure detailed by Malmberg [29] to study taxonomic features of the haptor (= opisthaptor, or terminal attachment organ), male copulatory organ (MCO) and pharynx. Further specimens had their haptors excised using a scalpel and were subjected to proteolytic digestion as described previously [30], to release the attachment hooks from enclosing tissue. The corresponding anterior portions of bisected bodies were stored at -20 °C in 96% ethanol, individually labelled for subsequent molecular analyses. The hooks were mounted in a semi-permanent 1:1 formalin : glycerin solution and the edges of the coverslip were then sealed with the permanent mounting medium Pertex (Histolab Products AB, Gothenburg, Sweden).

### Morphological analysis

For the morphological study, images of the haptoral attachment hooks of proteolytically-digested specimens were captured using a Zeiss AxioCam MRc digital camera interfacing with an Olympus BH2 compound microscope using a ×0.75 lens and MRGrab 1.0.0.4 (Carl Zeiss Vision GmbH, 2001) software. Each gyrodactylid specimen was subjected to morphometric analysis taking 24 point-to-point measurements on the haptoral hooks using a JVC KY–F30B 3CCD video camera mounted on an Olympus BH2 microscope using a ×2.5 interfacing lens at ×100 oil immersion and the gyrodactylid-specific Point-R macro (Bron & Shinn, University of Stirling) written within the KS300 (ver.3.0) (Carl Zeiss Vision GmbH, 1997) image analysis software. The 24 morphometric measurements are given in micrometres or in degrees (for angles) as the range followed by the mean in parentheses, and were selected from those described in Malmberg [29], Shinn et al. [31] and Paladini et al. [32]. Holotypes and voucher specimens of *G. lamothei* (CNHE 6310, 6749-6753 and 7120) and *G. mexicanus* (CNHE 6745-6749 and 7128) were borrowed from the Colección Nacional de Helmintos (CNHE), Mexico City, and analysed morphometrically.

To describe and compare the aspect ratio of the hamuli of different *Gyrodactylus* spp., the quotient between the mean hamulus proximal shaft width (HPSW) and the hamulus total length (HTL) was calculated. Measurements used to calculate the aspect ratio of *G. lamothei*, *G. mexicanus* and those of gyrodactylids recovered from *G. atripinnis* are new. Morphometric data of *Gyrodactylus* spp. infecting poeciliid fishes were taken from [25] and [14].

### Molecular analyses

Individual genomic DNA of 17 ethanol-fixed bodies of excised specimens of *Gyrodactylus* spp. collected from *G. atripinnis*, and of three worms collected from *G. multiradiatus* were extracted using a DNeasy® Blood & Tissue Kit (Qiagen, Valencia, California, USA) following the manufacturer’s instructions. The target region spanning the 3′ end of the 18S rRNA gene, ITS1, 5.8S rRNA gene, ITS2 and the 5′ end of the 28S rRNA gene subunit failed to amplify using the primers employed previously by Rubio-Godoy et al. [33]. New primers ITS1-fm 5′-TAG AGG AAG TAC AAG TCG-3′ and ITS2-rm 5′-CGC TYG AAT CGA GGT CAG GAC-3′ were designed to facilitate amplification of the target using the following PCR conditions: initial denaturation for 4 min at 95 °C followed by 35 cycles of: 94 °C for 30 s, 54 °C for 45 s, 72 °C for 1 min, with a final extension at 72 °C for 7 min. PCR amplicons were visualised on GelRed (Biotium, San Francisco, California, USA) stained 1% agarose gels and then purified with ExoSap-IT (USB Corporation, Cleveland, Ohio, USA). Sequencing reactions were carried out with the use of Big Dye Terminator chemistry, incorporating the same primers as those used in PCR, and cleaned by filtration with Sephadex G50. The sequenced products were read on an ABI PRISM 3100 automated DNA sequencer (Applied Biosystems, Foster City, California, USA). Electropherograms were visually inspected with the use of FinchTV (Geospiza Inc., Seattle, Washington, USA), and overlapping fragments of forward and reverse sequences were assembled with the use of the computer program BioEdit v. 7.0.9 [34]. Sequences generated in the present study were deposited in the GenBank database.

New sequences of the ITS1, 5.8S rRNA gene, and ITS2 of the *Gyrodactylus* spp. infecting goodeid fishes were compared with the following *Gyrodactylus* sequences available from GenBank: *Gyrodactylus actzu* García-Vásquez, Razo-Mendivil & Rubio-Godoy, 2015; *Gyrodactylus apazapanensis* García-Vásquez, Razo-Mendivil & Rubio-Godoy, 2015; *Gyrodactylus arcuatus* Bychowsky, 1933; *G. bullatarudis*; *Gyrodactylus gondae* Huyse, Malmberg & Volckaert, 2004; *Gyrodactylus jarocho* Rubio-Godoy, Paladini, García-Vásquez & Shinn, 2010; *Gyrodactylus lhkauhili* García-Vásquez, Razo-Mendivil & Rubio-Godoy, 2015; *Gyrodactylus microdactylus* García-Vásquez, Razo-Mendivil & Rubio-Godoy, 2015; *Gyrodactylus pakan* Razo-Mendivil, García-Vásquez & Rubio-Godoy, 2016; *Gyrodactylus pictae* Cable, van Oosterhout, Barson & Harris, 2005; *Gyrodactylus poeciliae* Harris & Cable, 2000; *G. pseudobullatarudis*; *Gyrodactylus stephanus* Mueller, 1937; *Gyrodactylus takoke* García-Vásquez, Razo-Mendivil & Rubio-Godoy, 2015; *Gyrodactylus teken* Razo-Mendivil, García-Vásquez & Rubio-Godoy, 2016; *Gyrodactylus turnbulli* Harris, 1986; *Gyrodactylus xalapensis* Rubio-Godoy, Paladini, García-Vásquez & Shinn, 2010; and *G. xtachuna*. All sequences were aligned using MUSCLE v. 3.5 [35], implemented in the software SEAVIEW v. 4.2 [36]. Phylogenetic hypotheses of ITS sequences were inferred with maximum likelihood (ML) and Bayesian inference (BI). Prior to ML and BI analyses, the optimal model of evolution and parameter settings for the ITS dataset was selected in jModeltest 2.1.10 [37, 38] using the Akaike information criterion (AIC) [39] and the TVM+I+G model was selected. The likelihood program GARLI 2.0 [40] was used for topology reconstruction under a GTR model, allowing the program to estimate the I and G parameters. Two independent likelihood analyses were conducted for each data set to ensure convergence. Analyses were terminated after 100,000 generations, with an additional 1000 bootstrap replicates used for evaluating nodal support; each replicate was terminated after 10,000 generations. Bayesian phylogenetic relationships were inferred using MrBayes v.3.2.1 [41], running 10,000,000 generations and sampling one tree every 100 generations. Parameter settings used were nst = 6 and rates = invgamma. Posterior probabilities for supported clades were determined by a 50% majority-rule consensus of the 180,002 trees retained after 10% ‘burn-in’. Trees resulting of ML and BI analyses were rooted with *G. pakan* and *G. teken*, parasites of *Astyanax aeneus*, which occur sympatrically with poeciliids in their native distribution range in Veracruz. Finally, estimates of evolutionary divergence between ITS1, 5.8S and ITS2 sequences (number of base substitutions per site between sequence pairs) among species of *Gyrodactylus* parasitising goodeid and poeciliid fishes were obtained with MEGA7 [42]. All positions containing gaps and missing data were eliminated; there was a total of 677 positions in the final dataset.

## Results

Goodeid and poeciliid fishes were found to be sympatric in several locations in both the Atlantic Ocean and Pacific Ocean watersheds of Mexico. In general, invasive poeciliids were more numerous than endemic goodeids at localities sampled in the MC (Table 3). In the current study, only cases where gyrodactylids were recovered from blackfin goodea, *G. atripinnis* in localities where both goodeid and poeciliid fishes were collected simultaneously are presented (Table 3). As reported previously [14], poeciliids (*P. mexicana*) captured within their native distribution range in the Río Pánuco basin on the Gulf of Mexico slope were infected with *G. pseudobullatarudis* and *G. xtachuna*. None of the gyrodactylid species known to infect poeciliids were found on invasive fishes at the localities sampled within the MC (Table 3). Nonetheless, two invasive poeciliid species (*P. mexicana* and *P. bimaculatus*) collected at Araro, Michoacán, within the Río Lerma basin were infected with the cichlid fish pathogen *G. cichlidarum* (García-Vásquez A, Razo-Mendivil U, Rubio-Godoy M, unpublished). Further, undescribed species of *Gyrodactylus* were found infecting poeciliid fishes on localities draining into both sides of the continental divide (Table 2); these will be described following collection of further samples. Specimens of *G. lamothei* were recovered from the goodeid *G. multiradiatus* collected at San Nicolás Peralta, Estado de México, a locality within the MC in the Río Lerma basin. No specimens of *G. mexicanus* were recovered.

**Fig. 1:**
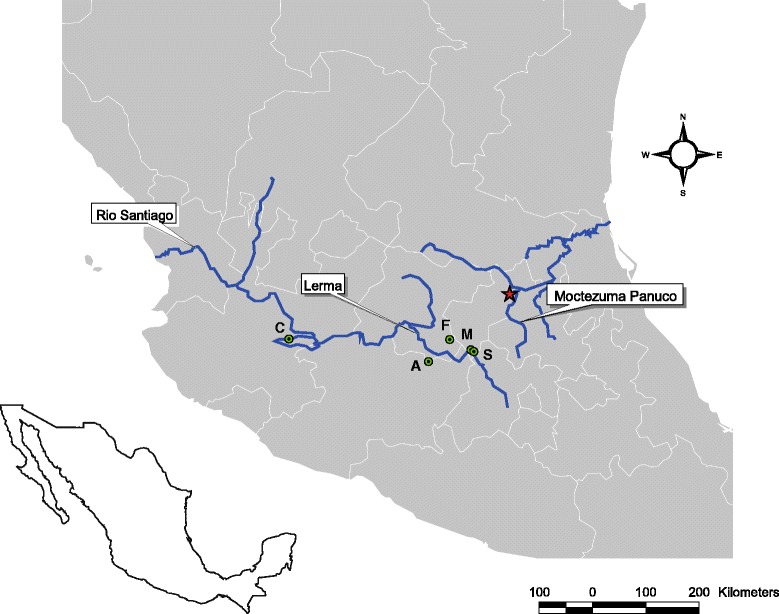
Map of Central Mexico, showing locations where *Gyrodactylus tomahuac* n. sp. was collected. The *red star* shows the type-locality of *G. tomahuac* n. sp. at Vega de Ramírez, Río Moctezuma, Querétaro (Qro). Río Moctezuma is a tributary of the Río Pánuco (shown in *blue*), the only major river from the Mesa Central (MC) that drains into the Gulf of Mexico. Locations where *G. tomahuac* n. sp. and other gyrodactylids were collected from *Goodea atripinnis* in the Río Lerma-Santiago basin, which drains the MC into the Pacific Ocean, are shown with letters: *A*, Araro, Michoacán; *C*, Lago de Chapala, Jalisco; *F*, El Fresno, Guanajuato; *M*, San Miguel Tlaxcaltepec, Qro; *S*, Santiago Mezquititlán, Qro

Morphological and molecular analyses indicated that four undescribed species of *Gyrodactylus* infect the blackfin goodea *G. atripinnis* (Table 3). In the current study, only one species is described, i.e. the most abundant and geographically widespread gyrodactylid species, which was collected from both sides of the continental divide (Fig. 1). The other three undescribed species of *Gyrodactylus* (referred to as *Gyrodactylus* sp. 1, sp. 2 and sp. 3) were found in limited numbers (i.e. 1, 2 and 4 specimens, respectively) and in only a few localities (for details see Table 3); these latter species are not described taxonomically in the present work. The findings of the study, however, broadly illustrate that the three undescribed species differ morphologically from other known gyrodactylids infecting goodeid and poeciliid fishes; and molecular data obtained from them are presented, because given the scarcity of genetic data on Mexican *Gyrodactylus* spp., these new sequences inform the phylogenetic relationships between gyrodactylid parasites infecting goodeid and poeciliid fishes. A summary of the specimens prepared from each locality and analysed by morphological and molecular means is presented in Table 3.

### *Gyrodactylus tomahuac* n. sp.

***Type-host***: *Goodea atripinnis* Jordan (“blackfin goodea”, “Tiro”) (Cyprinodontiformes: Goodeidae).

***Type-locality***: Río Moctezuma (21°03′31.01″N, 99°28′03.68″W), at Vega de Ramírez, Cadereyta de Montes, Querétaro, Mexico.

***Other localities***: Streams in Araro, Michoacán (19°54′27.52″N, 100°50′23.36″W); El Fresno, Guanajuato (20°16′39.07″N, 100°29′09.69″W); and San Miguel Tlaxcaltepec, Querétaro (20°06′23.34″N, 100°07′36.74″W).

***Type-material***: Fifteen specimens were studied for light microscopy, nine from the type-locality and six from other localities (Table 2). Holotype (CNHE accession no. 9991), paratype (CNHE no. 9992) and voucher specimens (CNHE nos. 9930 through 9932, and 9993 through 9995) are deposited in the Colección Nacional de Helmintos, Instituto de Biología, Universidad Nacional Autónoma de México (UNAM), Mexico City, Mexico. In addition, three voucher specimens (CMNPA accession no. 2015-0009, CMNPA 2015-0010 and CMNPA 2015-0011) have been deposited in the Canadian Museum of Nature, Parasite Collection (CMNPA), Ontario, Canada.

***Site on host***: Fins.

***Representative DNA sequences***: Partial ITS1 and ITS2 sequences and complete 5.8S ribosomal RNA gene sequences, with a length of 904 bp, have been deposited in GenBank under accession numbers KJ621983 (type-locality specimen, *n* = 1) and KR815847–KR815852 (Río Lerma basin paratypes/voucher specimens, *n* = 6).

***ZooBank registration***: In accordance with the regulations set out in article 8.5 of the *International Code of Zoological Nomenclature* (ICZN) [43], details of the new species have been submitted to ZooBank. The Life Science Identifier (LSID) of the article is urn:lsid:zoobank.org:pub:375D2AD9-11F8-4705-B966-A1EA19C2CA5B. The LSID for the new name *Gyrodactylus tomahuac* is urn:lsid:zoobank.org:act:73980450-57E3-4A03-9F17-70BD0E392ECB. The electronic edition of this work is published in a journal with an ISSN, and has been archived and is available from the following digital repositories: PubMed Central, LOCKSS. In addition, a species profile including taxonomic traits, host details and additional metadata is provided on www.gyrodb.net [44].

***Etymology***: From the Náhuatl (Aztec) word “*tomahuac*” meaning thick, robust, which describes the nature of the hamuli.

### Description

**Fig. 2:**
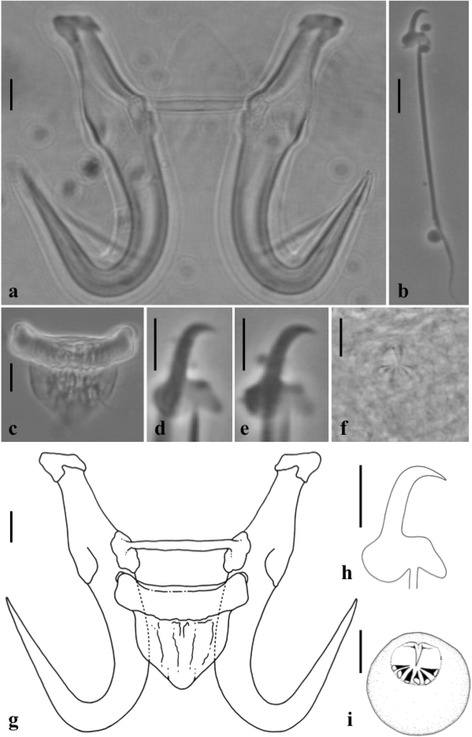
Haptoral armature and male copulatory organ of *Gyrodactylus tomahuac* n. sp. ex *Goodea atripinnis*. **a** Hamuli and dorsal bar. **b** Marginal hook. **c** Ventral bar. **d**-**e** Marginal hook sickles. **f** Male copulatory organ (MCO). **g** Line drawing of the hamulus complex. **h** Line drawing of the marginal hook sickle. **i** Line drawing of the MCO. *Scale-bars*: **a**-**c**, **g**, 5 μm; **d**-**f**, **h**-**i**, 3 μm

[Body measurements based on six whole worms, partially proteolytic-digested ammonium picrate glycerine, coverslip-flattened specimens (i.e. not processed for morphometric analysis of haptoral structures); see Table 4.] Body 223–345 (273) long, 70–76 (73.7) wide at uterus mid-point. Anterior pharyngeal bulb 16 × 24, posterior pharyngeal bulb 10 × 30. Excretory bladders present. Gut not extending beyond posterior terminus of uterus, almost reaching limit of haptor; testes and ovary not discerned. Haptor circular, clearly delineated from body, 68 × 63. Male copulatory organ posterior to pharynx, visible on one specimen only, 7.9 × 9.3, armed with a single curved apical spine facing a single row of 6 equally sized spines, 1.8 long (Fig. 2f, i). Hamuli, proportionately stout (Fig. 2a, g); total length 42.2–46.4 (44.9); broad at dorsal bar attachment point with proximal shaft width of 4.0–5.1 (4.4); shaft length 24.9–26.4 (25.6) long; similarly-sized point 22.3–24.5 (23.5) long such that each hamulus tip extends towards ventral bar articulation point on each hamulus, creating small hamulus aperture distance 10.9–15.0 (12.6) long and narrow aperture angle of 25.5–36.1° (30°). Root portion of each hamulus narrows away from shaft (i.e. from dorsal and ventral bar attachment points) with both dorsal and ventral surfaces of root, 17.8–21.5 (19.3) long, following a shallow, recurved line. Root terminus of each hamulus slopes towards its anterior and inner extremity. Dorsal bar attachment points approximately rectangular; dorsal bar simple, 18.7–21.6 (20.2) long, attached to upper third of dorsal bar attachment point; bar broadens slightly away from its attachment to each hamulus and then narrows marginally at its mid-point, 1.8–2.3 (2.0) wide (Fig. 2a). Anterior edge of median portion of ventral bar proper straight, posterior edge curves gently (Fig. 2c). Ventral bar processes small, rounded, 1.6–2.5 (2) long, positioned laterally in anterior third of ventral bar extremities. Ventral bar membrane lingulate at its anterior portion, posterior portion ends as a narrow “V”, 9.2–14.4 (10.7) long. Total length of marginal hooks 29.5–31.7 (31.0) (Fig. 2b); marginal hook shaft 24.0–26.7 (25.9) long; marginal hook sickle proper 5.2–5.8 (5.5) long. Shaft of sickle proper, angled approximately forward 15° to the perpendicular, uniform along its length, before turning almost 90° into a short tip that terminates at a point in line with approximate anterior third of sickle toe (Fig. 2d, e, h). Sickle distal width 2.0–2.7 (2.3), proximal width 3.7–4.4 (4.0). Aperture of marginal sickle, 4.5–5.3 (4.9) wide, inner curve of sickle proper approximately rectangular. Base of sickle proper with a flat bridge, approximately one third the proximal width of base; triangular toe 1.2–1.8 (1.6) long, terminates below marginal hook shaft insertion point on sickle proper and in line with posterior edge of heel. Sickle instep height 0.4–0.8 (0.6). Heel of sickle proper, pronounced, approximately rhomboid in dimensions and drops 35° in a downwards direction from the perpendicular (Fig. 2d, e, h).

**Table 4:** Morphological measurements of *Gyrodactylus tomahuac* n. sp. from *Goodea atripinnis* Jordan collected in Mexico

Measurement	*G. tomahuac* n. sp. Río Pánuco basin, type-locality (*n* = 9)		*G. tomahuac* n. sp. Río Lerma basin (*n* = 6)	
	Range	Mean	Range	Mean
Hamulus				
Total length	42.2–46.4	44.9	44.5–48.3	46.4
Shaft length	24.9–26.4	25.6	26.3–28.9	27.3
Point length	22.3–24.5	23.5	24.9–27.1	26.2
Root length	17.8–21.5	19.3	18.8–22.5	20.0
Proximal shaft width	6.6–8.5	7.8	8.3–8.8	8.5
Aperture angle (^o^)	25.5–36.1	30.0	28.0–31.1	29.8
Aperture distance	10.9–15.0	12.6	12.9–14.3	13.3
Distal shaft width	4.0–5.1	4.4	4.3–5.1	4.7
Ventral bar				
Total length	15.6–21.0	18.5	16.6–20.9	19.2
Total width	18.4–21.9	20.5	21.5–23.0	22.2
Process-to-mid length	2.0–3.5	2.8	2.2–4.0	2.9
Median length	4.7–5.9	5.4	5.4–6.1	5.8
Process length	1.6–2.5	2.0	0.9–3.3	1.8
Membrane length	9.2–14.4	10.7	10.0–12.2	11.0
Dorsal bar				
Total length	18.7–21.6	20.2	17.9–22.0	20.1
Width	1.8–2.3	2.0	1.6–2.4	2.0
Marginal hook				
Total length	29.5–31.7	31.0	29.6–31.1	30.3
Shaft length	24.0–26.7	25.9	25.1–27.0	25.9
Sickle length	5.2–5.8	5.5	4.8–6.1	5.4
Sickle proximal width	3.7–4.4	4.0	3.5–4.3	3.9
Toe length	1.2–1.8	1.6	1.6–1.9	1.7
Sickle distal width	2.0–2.7	2.3	2.0–2.8	2.4
Aperture	4.5–5.3	4.9	4.6–5.2	4.9
Instep/arch height	0.4–0.8	0.6	0.4–1.0	0.7

### Remarks

*Gyrodactylus tomahuac* n. sp. is the third gyrodactylid described from *G. atripinnis*. This host has previously been found to harbour *G. lamothei* and *G. mexicanus* [24], as well as undescribed species of *Gyrodactylus* (Table 2). At the type-locality, Vega de Ramírez, Río Moctezuma, Querétaro, the only gyrodactylid species recorded was *G. tomahuac* n. sp., with 41 parasites found on ten hosts (Table 3). At other localities, *G. tomahuac* n. sp. burdens were lower; and *G. tomahuac* n. sp. was found concurrently infecting the same hosts as *Gyrodactylus* sp. 3 in two localities in the Río Lerma basin (Table 3). *Gyrodactylus tomahuac* n. sp. was found in four different localities in the Mexican highlands (Fig. 1) at considerable distances from each other: Vega de Ramírez, Río Moctezuma is *c*.150 km apart from the other three locations in the Río Lerma basin where the parasite was recorded (i.e. El Fresno, San Miguel Tlaxcaltepec, and Araro), and these three sites are *c*.60 km apart from each other. More importantly, the parasite was recorded in river basins flowing into both directions of the continental divide: the type-locality (Vega de Ramírez) at the Río Moctezuma is within the Río Pánuco basin, which drains into the Gulf of Mexico; while streams at El Fresno, San Miguel Tlaxcaltepec and Araro are part of the Río Lerma-Santiago basin and flow into the Pacific Ocean.

**Fig. 3:**
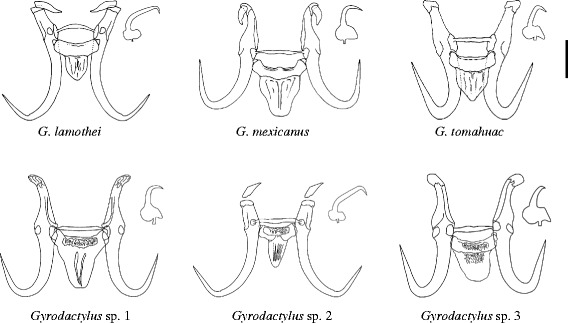
Haptoral armatures of different species of *Gyrodactylus* von Nordmann, 1832 infecting goodeid fishes. *Gyrodactylus* sp. 1, sp. 2, and sp. 3 refer to the three undescribed but genetically characterised species reported in this work. *Scale-bar*: 10 μm

**Fig. 4:**
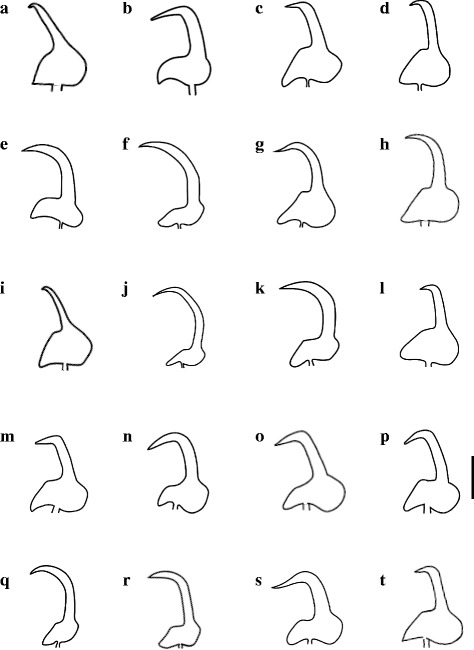
Marginal hook sickles of different species of *Gyrodactylus* von Nordmann, 1832 infecting poeciliid fishes. The marginal hook of *Gyrodactylus tomahuac* n. sp., a parasite of the goodeid fish *Goodea atripinnis* is shown for comparison. **a** *G. actzu.* **b** *G. apazapanensis.* **c** *G. bullatarudis.* **d** *G. costaricensis.* **e** *G. cytophagus.* **f** *G. gambusiae.* **g** *G. lhkahuili.* **h** *G. jarocho.* **i** *G. microdactylus*. **j** *G. milleri.* **k** *G. pictae*. **l** *G. poeciliae.* **m** *G. pseudobullatarudis.* **o** *G. takoke.* **p** *G. tomahuac* n. sp. **q** *G. turnbulli.* **r** *G. unami.* **s** *G. xalapensis.* **t** *G. xtachuna. Scale-bar*: 5 μm

The morphology of the marginal hooks is considered the most informative character to differentiate between species of *Gyrodactylus*. The marginal hooks of *G. tomahuac* n. sp. clearly differ in shape from those of both *G. lamothei* and *G. mexicanus* (Fig. 3): the marginal hooks of *G. lamothei* possess a very long and forward-tilted shaft whose tip ends well beyond the toe of a rounded, almost elliptical base, in contrast to *G. tomahuac* n. sp. where the marginal hook shaft is upright and does not extend beyond the angular base of the toe; and the marginal hooks of *G. mexicanus* have an upright shaft ending in a downward-curved point almost as long as the shaft itself, and the marginal hook base has an extended bridge ending in a rounded toe, in contrast to the comparatively short shaft point and short-bridged, angular toe in *G. tomahuac* n. sp. Superficially, the marginal hooks of *G. tomahuac* n. sp. resemble those of two gyrodactylids infecting poeciliid fishes, *G. bullatarudis* and *G. takoke* (Fig. 4); however, features of the marginal hook base allow their discrimination. While the marginal hook base in *G. tomahuac* n. sp. has a clear bridge almost level with the union of the heel and the shaft which forms a sharp angle with an almost straight, downward inclined toe, in both *G. bullatarudis* and *G. takoke* the toe curves gently downward and there is only a very short bridge in the first, and no bridge in the latter.

**Fig. 5:**
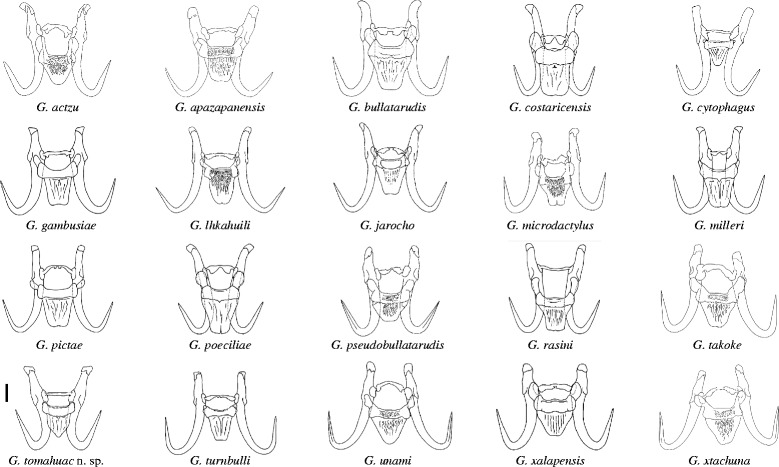
Hamulus complex, including dorsal and ventral bars of different species of *Gyrodactylus* von Nordmann, 1832 infecting poeciliid fishes. The hamulus complex of *Gyrodactylus tomahuac* n. sp., a parasite of the goodeid fish *Goodea atripinnis* is shown for comparison. *Scale-bar*: 10 μm

*Gyrodactylus tomahuac* n. sp. can also be differentiated from gyrodactylids known to infect goodeid and poeciliid fishes, comparing the overall morphology of their haptoral sclerites. Based on the variation in the haptoral hook morphology of the *Gyrodactylus* species infecting goodeid fishes (Fig. 3), two groups can be roughly identified: *G. lamothei* and *Gyrodactylus* sp. 2 both have relatively slender hamuli and marginal hooks with forward-angled shafts extending well beyond the toe, while *G. tomahuac* n. sp., *G. mexicanus* and both *Gyrodactylus* sp. 1 and sp. 3 possess robust hooks, with comparatively wider hamulus proximal shafts (as shown by the ratio between the mean hamulus proximal shaft width (HPSW) to the hamulus total length (HTL; Table 5), and marginal hooks with upright, shorter sickle shafts. Further, *G. tomahuac* n. sp. can easily be distinguished from *G. lamothei*, *G. mexicanus* and *Gyrodactylus* sp. 2, as these have elongated sclerotised structures attached to the root of the hamuli (Fig. 3). The haptoral hook morphology of *G. tomahuac* n. sp. is quite distinctive from that of most gyrodactylid species infecting poeciliid fishes (Fig. 5), except for perhaps *G. bullatarudis*, *G. gambusiae* Rogers & Wellborn, 1965 and *G. pictae*, all of which have robust hamuli (Table 5); albeit these species can be easily separated from *G. tomahuac* n. sp. because they all have longer hamuli (HTL > 50 μm *vs c*.45 μm) and since the ventral bars of these gyrodactylids infecting poeciliid fishes are comparatively larger and have long processes, which are absent on the relatively smaller ventral bar of *G. tomahuac* n. sp. Some other gyrodactylids infecting poeciliids have stout hamuli (HPSW/HTL ratio = 0.16; Table 5), but the majority possess slender hamuli.

**Table 5:** Aspect ratio of the hamuli of *Gyrodactylus* spp. infecting goodeid and poeciliid fishes

Fish host	*Gyrodactylus* spp.	HPSW	HTL	Ratio HPSW/HTL^a^	Mean HTL	Mean ratio	Aspect
*G. atripinnis*	*G. tomahuac* n. sp. (type-locality)	7.8	44.9	0.17			Robust
	*G. tomahuac* n. sp. (Lerma basin)	8.5	46.4	0.18	45.650	0.178	Robust
*G. atripinnis*	*Gyrodactylus* sp. 1	9.3	56.6	0.16			Stout
*G. atripinnis*	*Gyrodactylus* sp. 2	8.6	56.6	0.15			Slender
*G. atripinnis*	*Gyrodactylus* sp. 3	9.8	62.3	0.16	58.500	0.158	Stout
*G. multiradiatus*	*G. lamothei*	6.9	46.6	0.15			Slender
	*G. mexicanus*	7.8	46.6	0.17			Robust
Poeciliid fishes	*G. actzu*	7.8	52.9	0.15			Slender
	*G. apazapanensis*	9	57.2	0.16			Stout
	*G. bullatarudis*	9.4	56	0.17			Robust
	*G. costaricensis*	11.7	71.7	0.16			Stout
	*G. cytophagus*	7.6	54.2	0.14			Slender
	*G. gambusiae*	9.9	50.2	0.20			Robust
	*G. jarocho*	8.6	67.1	0.13			Slender
	*G. lhkauhuili*	8.4	67.5	0.12			Slender
	*G. microdactylus*	8.3	52.7	0.16			Stout
	*G. milleri*	8.1	56.2	0.14			Slender
	*G. pictae*	9.2	55.2	0.17			Robust
	*G. poeciliae*	7.9	58.8	0.13			Slender
	*G. pseudobullatarudis*	7.8	51.3	0.15			Slender
	*G. rasini*	7.3	50.7	0.14			Slender
	*G. takoke*	7.7	47.6	0.16			Stout
	*G. turnbulli*	8.7	57.9	0.15			Slender
	*G. unami*	6.5	45.6	0.14			Slender
	*G. xalapensis*	6.8	47.2	0.14			Slender
	*G. xtachuna*	7.7	53.9	0.14	55.468	0.151	Slender

Note: Measurements of *G. lamothei* and *G. mexicanus* are new. Morphometric data of *Gyrodactylus* spp. infecting poeciliid fishes taken from Rubio-Godoy et al. [25] and García-Vásquez et al. [14]

*Abbreviations*: *HPSW* hamulus proximal shaft width, *HTL* hamulus total length

^a^Informal aspect categorisation scale for Ratio HSPW/HTL: Robust (> 0.17), Stout (= 0.16), Slender (< 0.15)

### Phylogenetic analyses

The length of the new ITS sequences of 14 specimens of *Gyrodactylus* spp. (seven *G. tomahuac* n. sp., one *Gyrodactylus* sp. 1, two *Gyrodactylus* sp. 2, and four *Gyrodactylus* sp. 3) collected from *G. atripinnis* varied from 866 to 934 bp. ITS sequences obtained for *G. lamothei* were deposited in GenBank (GenBank acc. nos. KX555666–KX555668), and varied from 887 to 888 bp. One representative sequence of each of these mentioned taxa was compared with those downloaded from GenBank, producing an alignment composed by 24 sequences with a length of 1324 bp. Phylogenetic analyses did not include *G. mexicanus*, because no ITS sequences are available in GenBank for this species and we did not recover any specimens during the current survey.

**Fig. 6:**
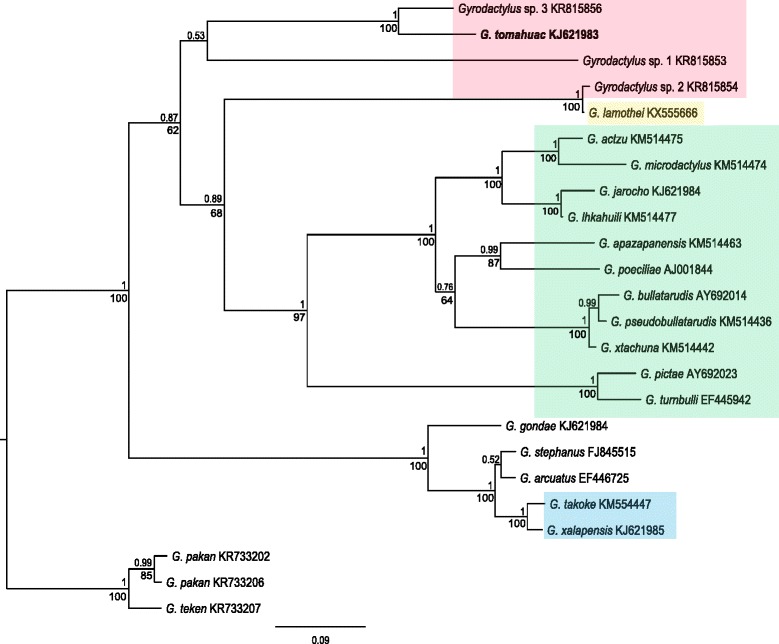
Phylogenetic tree of different *Gyrodactylus* species infecting teleost fishes. *Shaded boxes* indicate parasite clades infecting the following host groups: *Goodea atripinnis* (*red*), other goodeid fishes (*yellow*), and poeciliid fishes (*green* and *blue*). Fifty percent majority-rule consensus tree obtained from the Bayesian Inference analysis of the ITS1, 5.8S and ITS2 sequences from 23 species of *Gyrodactylus*. Bayesian posterior probabilities are shown above the nodes, and ML bootstrap support below the nodes. The phylogram is rooted with gyrodactylids infecting the characid *Astyanax aeneus*

ML and BI analyses of ITS sequences recovered identical phylogenetic relationships among the analysed species, with several well-supported nodes. ML analysis resulted in one tree (not shown) with a value of likelihood = -10,210.6007. The 50% majority-rule consensus tree (Fig. 6) obtained from the BI analysis recovered three main clades, in which the sequences of *Gyrodactylus* spp. infecting goodeid and poeciliid fishes do not constitute monophyletic assemblages; and the gyrodactylids from goodeid fishes appeared in two clades constituting five different lineages.

Sequences of *G. tomahuac* n. sp., *Gyrodactylus* sp. 1 and *Gyrodactylus* sp. 3 formed a main clade with very low branch support (0.5321 of pp). This clade is composed by one terminal branch (*Gyrodactylus* sp. 1) and one derived group containing *G. tomahuac* n. sp. and *Gyrodactylus* sp. 3 (Fig. 6; red box). A second main clade contains two subclades, one of them constituted by the goodeid-infecting parasites *Gyrodactylus* sp. 2 and *G. lamothei* (red and yellow boxes), which appear as sister group to a cluster of mostly well-supported derived branches containing 11 species of *Gyrodactylus* infecting poeciliid fishes (green box). Finally, a third main clade has one terminal and two derived branches containing parasites infecting several non-related fish hosts: *G. gondae* infecting Lozano’s goby *Pomatoschistus lozanoi* (de Buen); *G. stephanus* infecting common mummichogs *Fundulus heteroclitus* (L.); *G. arcuatus* infecting three-spined sticklebacks *Gasterosteus aculeatus* L.; and two gyrodactylids from poeciliids, *G. xalapensis* and *G. takoke* (blue box).

### Intra- and inter-specific nucleotide variation

Comparison of ITS1, 5.8S and ITS2 sequences among the 24 species analysed showed a great amount of nucleotide variation, ranging from 0.4 to 32.7%. The species pairs that showed these contrasting values were, respectively, *G. lamothei* and *Gyrodactylus* sp. 2, and both *G. arcuatus* and *Gyrodactylus* sp. 2, as well as *G. jarocho* and *G. stephanus*. Likewise, new sequences of the gyrodactylids collected from *G. atripinnis*, representing *G. tomahuac* n. sp. and three unnamed species (*Gyrodactylus* sp. 1, sp. 2, and sp. 3) exhibited high sequence variation (Table 6). Nucleotide differences ranging from 6.8 to 31.3% were found among the 14 sequences of *Gyrodactylus* spp. infecting this goodeid fish. Values of nucleotide variation between the new species, *G. tomahuac*, and the other three unnamed species (*Gyrodactylus* sp. 1, sp. 2, and sp. 3) were 25.6 to 25.9%, 24.0 to 24.2%, and 6.8 to 7.1%, respectively.

**Table 6:** Uncorrected pairwise distance “*p*” (%) of ITS1 and ITS2 sequences among fourteen specimens of *Gyrodactylus* spp. infecting goodeid fishes

	Isolate	1	2	3	4	5	6	7	8	9	10	11	12	13	14
1	*G. tomahuac* n. sp. isolate 1	–													
2	*G. tomahuac* n. sp. isolate 2	0.13	–												
3	*G. tomahuac* n. sp. isolate 3	0.13	0	–											
4	*G. tomahuac* n. sp. isolate 4	0.27	0.13	0.13	–										
5	*G. tomahuac* n. sp. isolate 5	0.13	0	0	0.13	–									
6	*G. tomahuac* n. sp. isolate 6	0.13	0	0	0.13	0	–								
7	*G. tomahuac* n. sp. isolate 7	0.13	0	0	0.13	0	0	–							
8	*Gyrodactylus* sp. 1 isolate 1	25.6	25.8	25.8	25.9	25.8	25.8	25.8	–						
9	*Gyrodactylus* sp. 2 isolate 1	24.2	24.0	24.0	24.1	24.0	24.0	24.0	31.3	–					
10	*Gyrodactylus* sp. 2 isolate 2	24.2	24.0	24.0	24.1	24.0	24.0	24.0	31.3	0	–				
11	*Gyrodactylus* sp. 3 isolate 1	7.0	7.0	7.0	7.1	7.0	7.0	7.0	25.3	24.6	24.6	–			
12	*Gyrodactylus* sp. 3 isolate 2	6.8	6.8	6.8	6.9	6.8	6.8	6.8	25.1	24.5	24.5	0.13	–		
13	*Gyrodactylus* sp. 3 isolate 3	6.8	6.8	6.8	6.9	6.8	6.8	6.8	25.1	24.5	24.5	0.13	0	–	
14	*Gyrodactylus* sp. 3 isolate 4	7.0	6.9	6.9	7.1	6.9	6.9	6.9	25.3	24.6	24.6	0.12	0	0	–

Intra-specific nucleotide variation was detected in *G. tomahuac* n. sp. and in *Gyrodactylus* sp. 3. Of the seven sequenced specimens of *G. tomahuac* n. sp. and four *Gyrodactylus* sp. 3, four and one specimens showed nucleotide variation, which ranged from 0.13 to 0.27%, and from 0.12 to 0.13%, respectively. Intra-specific nucleotide variation ranging from 0.13 to 0.45% was detected in the three *G. lamothei* specimens sequenced.

## Discussion

The first objective of this study was to characterise the gyrodactylid fauna of *G. atripinnis* within its native distribution range in the MC; and in doing so, assess whether HS have occurred to this endemic host from alien poeciliid fishes, which have been extensively introduced to central Mexico.

No evidence was found of gyrodactylid HS from poeciliids to goodeids, nor vice versa, in the six localities where fishes of these families occurred sympatrically. In all instances, poeciliid fishes were more abundant than goodeid fishes (Table 3), and the former included species known to harbour several gyrodactylids: *P. mexicana* has the richest gyrodactylid fauna associated with poeciliids, as this host has been recorded to harbour six parasite species in Mexico; *P. bimaculatus* is a translocated species known to be infected by four gyrodactylid species; and the invasive *P. reticulata* has been registered as host to three gyrodactylids [14]. In their native Río Pánuco basin, *P. mexicana* were infected by *G. pseudobullatarudis* and *G. xtachuna*, two gyrodactylid species known to have a wide geographical and host range [14]. In the MC, poeciliid fishes were not infected by any of the gyrodactylids known to parasitise them in their native distribution range [14, 25], some of which have been widely disseminated through the ornamental aquarium trade [10, 13]. Although the current study was not designed to test the enemy release hypothesis, results suggest that upon translocation to the MC, poeciliid fishes have lost their gyrodactylid parasite fauna; however, this finding is based on the limited sample sizes of poeciliid fishes found sympatrically with *G. atripinnis* in a few localities only, and are by no means representative of invasive poeciliid populations established in the MC. Surprisingly, invasive *P. mexicana* and *P. bimaculatus* collected in the MC were found to carry the alien tilapia parasite *G. cichlidarum* (García-Vásquez A, Razo-Mendivil U, Rubio-Godoy M, unpublished).

*Goodea atripinnis* was found to be infected by *G. tomahuac* n. sp. and three undescribed *Gyrodactylus* spp. This host was previously recorded to be infected by *G. lamothei* and *G. mexicanum* [24], suggesting that this endemic fish is parasitised by at least six gyrodactylids. The helminth fauna of goodeid fishes is well-characterised [24], and *G. atripinnis* is the host species known to harbour most parasites: nine digeneans, five nematodes, four cestodes, three monogeneans (including *G. lamothei* and *G. mexicanum*) and one acanthocephalan are recorded to infect this host. Considering six gyrodactylid species as parasites of this host, monogeneans represent the second most numerous helminth group known to infect this endemic fish. Bearing in mind that several reports have been made of the presence of undescribed *Gyrodactylus* sp. infecting *G. atripinnis* and several other goodeid fishes (Table 2); and the fact that along with *G. multiradiatus*, *G. atripinnis* remains a common and relatively abundant fish in much of the MC [22], it is foreseeable that several new species of *Gyrodactylus* infecting goodeid fishes will be found, potentially making monogeneans the most species-rich group of helminths infecting this host group.

Regarding the characterisation of the gyrodactylid fauna of *G. atripinnis* and other goodeid fishes, both morphological and molecular data support the hypothesis that two distinct groups of parasites infect these native fishes. Morphologically, some gyrodactylids possess robust hamuli, while others have slender hamuli with sclerotised plates located at the base of these sclerites. Very robust hamuli are the most salient feature of *G. tomahuac* n. sp., the species described here, a characteristic shared with *G. mexicanus*, and to a slightly lesser degree with the undescribed *Gyrodactylus* sp. 1 and *Gyrodactylus* sp. 3, both of which have stout hamuli (Table 5). Both *G. lamothei* and the undescribed *Gyrodactylus* sp. 2 possess slender hamuli, with elongated plates located at their roots; these plates were also drawn in the description of *G. mexicanus* [26]. It would be interesting to ascertain whether these structures are similar to the sclerotised plates found in *Gyrodactylus proterorhini* Ergens, 1967 [45], and to establish the phylogenetic relationships of *G. mexicanus* to other gyrodactylids once molecular markers are available.

The phylogenetic tree presented here is the most complete hypothesis of the relationships between gyrodactylids infecting goodeid fishes; to date, the only molecular data available for these parasites are those presented in this work (Fig. 6). Based on this hypothesis, *Gyrodactylus* spp. infecting goodeid fishes do not constitute a monophyletic group, as these parasites appear in two clades, which correspond to the two groups formed based on the morphology of the hamuli. The first, not well-supported clade includes *G. tomahuac* n. sp. as sister species to the undescribed *Gyrodactylus* sp. 3, and *Gyrodactylus* sp. 1 as a terminal branch; these three taxa all have relatively robust hamuli, albeit their marginal hooks differ (Fig. 3). *Gyrodactylus lamothei* and *Gyrodactylus* sp. 2 both have slender hamuli with plates at their roots, and appear in a separate clade as sister taxa to 11 gyrodactylid species known to infect poeciliid fishes, most of which also possess slender hamuli. Two gyrodactylids known to infect poeciliid fishes, *G. takoke* and *G. xalapensis*, appear in a further clade, which includes parasites infecting a variety of non-related fish hosts. Molecular characterisation of further gyrodactylids infecting goodeid and poeciliid fishes should shed light on the phylogenetic hypotheses outlined here: that two morphologically-distinct parasite lineages infect goodeids, one of which shares a common ancestor with several gyrodactylids infecting poeciliids.

## Conclusions

No evidence was found of gyrodactylids switching host from introduced poeciliid fishes to endemic goodeid fishes in the MC; nor vice versa. *Goodea atripinnis* harbours at least six species of *Gyrodactylus*, including *G. tomahuac* n. sp., which is described here. Morphological and molecular data support the hypothesis that two groups of gyrodactylids infect goodeid fishes: one contains worms which possess robust hamuli including *G. tomahuac* n. sp., and the other encompasses parasites with slender hamuli and sclerotised plates at their roots such as *G. lamothei* - and phylogenetic analyses suggest that this second group shares a common ancestor with gyrodactylids infecting poeciliid fishes. Monogeneans may be the most species-rich group of helminth parasites infecting goodeid fishes.

## Abbreviations

AIC: Akaike information criterion
BI: Bayesian inference
CMNPA: Canadian Museum of Nature Parasite Collection, Ontario
CNHE: Colección Nacional de Helmintos Mexico City
HPSW: Hamulus proximal shaft width
HS: Host switch(es)
HTL: Hamulus total length
ITS: Internal transcribed spacer
MC: Mexican central highlands Mesa Central
MCO: Male copulatory organ
ML: Maximum likelihood
Qro: State of Querétaro
